# *COQ6* mutation in patients with nephrotic syndrome, sensorineural deafness, and optic atrophy

**DOI:** 10.1002/jmd2.12068

**Published:** 2020-05-05

**Authors:** R. Justine Perrin, Caroline Rousset‐Rouvière, Florentine Garaix, Aline Cano, John Conrath, Olivia Boyer, Michel Tsimaratos

**Affiliations:** ^1^ Assistance Public Hôpitaux de Marseille Service Multidisciplinaire Timone Marseille France; ^2^ Assistance Public Hôpitaux de Marseille Service de pédiatrie et Neurologie Marseille France; ^3^ Clinique Monticelli, Ophtalmologie Marseille France; ^4^ Hopital Necker Néphrologie Pediatrique Paris France

## Abstract

**Introduction:**

Primary coenzyme Q10 (CoQ10) deficiencies are a group of mitochondrial disorders that has proven responsiveness to replacement therapy. Mutations in enzymes involved in the biosynthesis of CoQ10 genes are associated with these deficits. The clinical presentation of this rare autosomal recessive disorder is heterogeneous and depends on the gene involved. Mutations in the *COQ2, COQ6, PDSS2*, and *ADCK4* genes are responsible for steroid‐resistant nephrotic syndrome (SRNS), which is associated with extra‐renal symptoms. Previous studies have reported *COQ6* mutations in 11 patients from five different families presenting with SRNS and sensorineural deafness.

**Case reports:**

Our study reports the cases of two brothers of Turkish origin with renal failure and sensorineural deafness associated with *COQ6* mutations responsible of CoQ10 deficiency. Optical symptoms were present in the eldest, that improved with Idebenone.

**Conclusion/Discussion:**

For the first time, *COQ6* mutation with optical involvement is associated with renal and hearing impairment. Although the response to replacement CoQ10 therapy was difficult to evaluate, we think that this treatment was able to stop the disease progression in both patients, and even to prevent the occurrence/development of optical and neurological impairment in the younger brother. Mitochondrial dysfunction secondary to CoQ10 deficiency should always be suspected in patients with SRNS and extra‐renal symptoms. Early recognition of this genetic SRNS is mandatory since SRNS can be avoided by adequate treatment based on CoQ10 supplement or an analogue. All cases of primary CoQ10 deficiency should be treated at an early stage to limit the progression of lesions and prevent the emergence of new symptoms.

**Abbreviations**
ACE: angiotensin‐converting enzyme
*ADCK4*: aarF domain containing kinase 4
*COQ2*: coenzyme Q2 4‐hydroxybenzoate polyprenyltransferase
*COQ6*: coenzyme Q 10 biosynthesis monooxygenase 6
COQ9: ubiquinone bisynthesis protein coenzyme Q9
CoQ10: coenzyme Q10
DMS: diffuse mesangial sclerosis
ESRF: end‐stage renal failure
FSGS: focal segmental glomerulosclerosis
NS: nephrotic syndrome
OCT: optical coherence tomography
*PDSS2*: prenyl (decaprenyl) diphosphate synthase subunit 2
SRNS: steroid‐resistant nephrotic syndrome
VA: visual acuity

## 1 INTRODUCTION

Nephrotic syndrome (NS), a chronic kidney disease, manifests with significant proteinuria, hypoalbuminemia, and edema. Approximately 20% of children with NS are typically resistant to steroids and other immunosuppressive therapy methods.^1^ Steroid‐resistant NS (SRNS) is a frequent cause of end‐stage renal failure (ESRF). SRNS represents a glomerular disease caused by numerous different etiologies, all of which lead to similar patterns of glomerular damage. In the majority of children with SRNS, light microscopy reveals focal segmental glomerulosclerosis (FSGS). Now that the single‐gene causes of SRNS have been identified, we have a deeper understanding of this disease's pathogenesis. Primary coenzyme Q10 deficiency is a rare but significant causative element in SRNS owing to it being the only treatable mitochondrial disorder. Coenzyme Q10 deficiency involves various mutations in different genes. Heeringa et al reported *COQ6* mutations in five Turkish and Lebanese families with SRNS and sensorineural deafness.^2^

This study reports the case of two Turkish siblings with renal impairment, sensorineural deafness, and optic atrophy exhibiting *COQ6* mutation. We sought to assess the efficacy of oral ubiquinone therapy in these patients.

## 2 CASE REPORTS

### 2.1 Patient 1

Patient 1 was the second child of a consanguineous Turkish couple, a boy born after a term pregnancy with normal delivery. No significant events were found in the family history. ESRF was diagnosed when the patient presented with asthenia, vomiting, and high blood pressure at 5 years old. Renal ultrasonography revealed normal‐sized kidneys with hyperechogenic cortex and normal renal Doppler imaging results. Renal biopsy detected lesions of pan nephritis with generalized glomerular fibrosis and skin biopsy revealed normal expression of the type IV collagen alpha chain. The patient started peritoneal dialysis immediately following diagnosis and underwent successful renal transplantation at 6 years old. He received immunosuppressive treatment with corticosteroids, azathioprine, and cyclosporine following anti‐lymphocyte serum administration. The short‐term evolution of transplantation was characterized by a primary cytomegalovirus infection. The long‐term outcome was good, with no rejection. In addition, this child presented with bilateral sensorineural deafness, diagnosed at 6 years old on account of his delay in language acquisition. Audiogram revealed a severe perception deficit in the high frequencies. Optical examination, by means of slit lamp, visual acuity, and fundus tests, was normal. The patient additionally exhibited normal psychomotor development.

**Figure 1:**
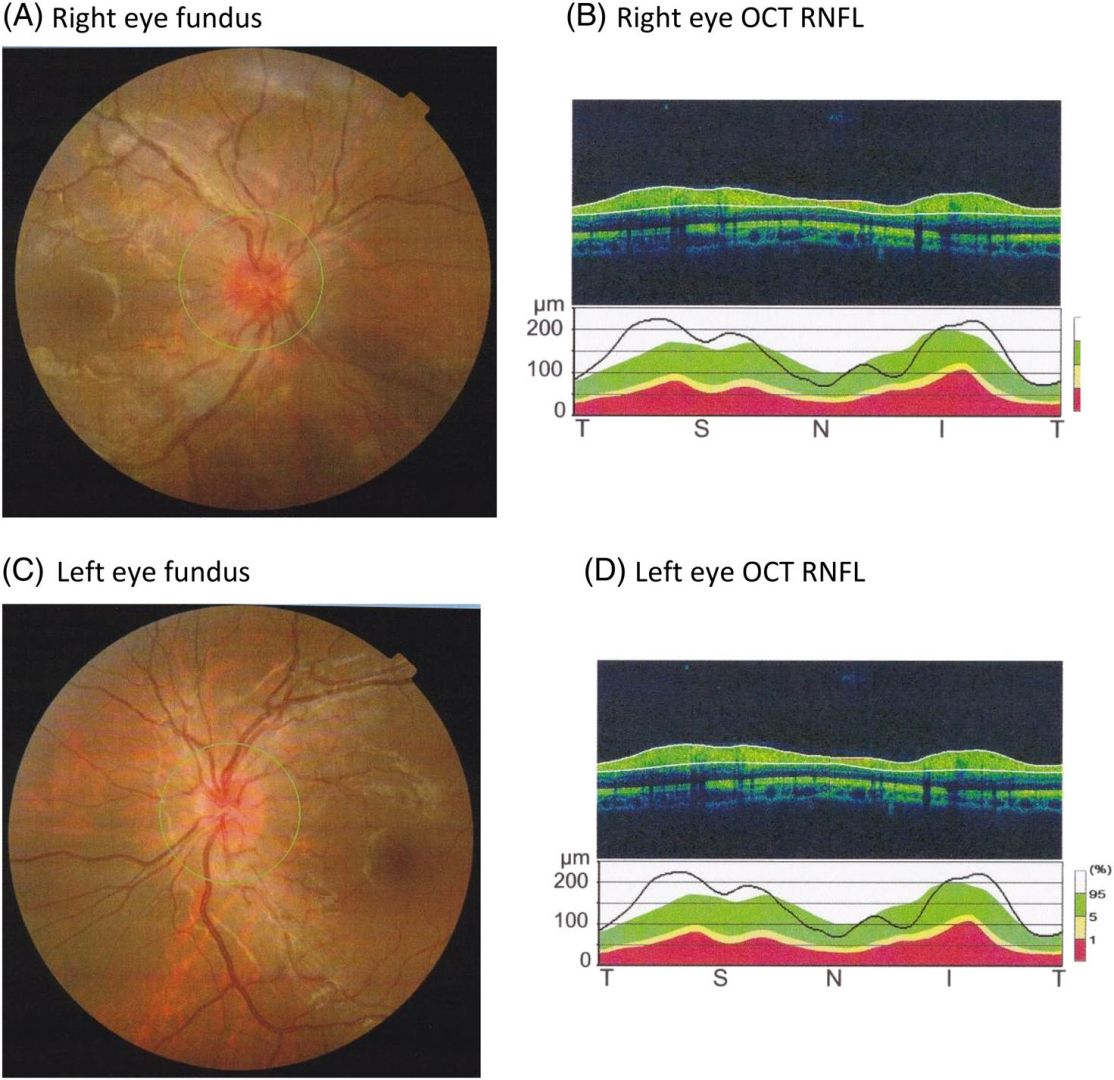
Optical examination in patient 1 before CoQ 10 supplementation. (A and C) Eye fundus. (B and D) optical coherence tomography (OCT) associated with an analysis of the retinal nerve fiber layer (RNFL). Bilateral papilledema

At 17 years, he complained of sudden visual loss. Optical examination revealed bilateral papilledema and a visual acuity (VA) of 20/200 for the right eye and 20/50 for the left (Figure 1). Optical coherence tomography (OCT), associated with an analysis of the retinal nerve fiber layer (RNFL) thickness, confirmed the existence of a bilateral papilledema. Inflammatory and infectious etiologies, were all ruled out. We conducted magnetic resonance imaging (MRI) of the brain and visual pathways, along with cerebral scan angiography, both coming back as negative for vasculitis lesions. Given the patient's severe optical involvement, and pending results, steroid therapy was initiated. The patient received four intravenous boluses of methylprednisolone (1 g/1.73 m^2^) then replaced with oral corticosteroids at 1 mg/kg/day for 10 days. Despite a slight decrease in papilledema, bilateral VA showed no improvement. The poor response to steroid treatment led us to suspect Leber's hereditary optic neuropathy. No mutation was found in the mitochondrial DNA. Exploration of the mitochondrial respiratory chain on the muscular biopsy was normal, including the combined activities of complexes I + III and II + III. Because of the ethnical backround and the association of renal and sensorineural symptoms we asked for molecular study of *COQ6* gene: genetic analysis detected a missense mutation c.1058C>A (p.Ala353Asp) in homozygous state in exon 9 of the *COQ6* gene. The same mutation was described by Heeringa et al in three patients from two families.^2^

**Figure 2:**
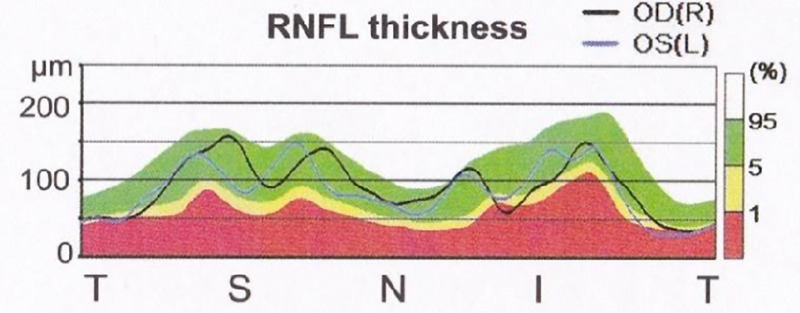
Optical examination in patient 1 after 2 months of CoQ 10 supplementation. Optical coherence tomography (OCT) associated with an analysis of the retinal nerve fiber layer (RNFL), right eye. Papilledema disappeared with onset of optic atrophy

**Figure 3:**
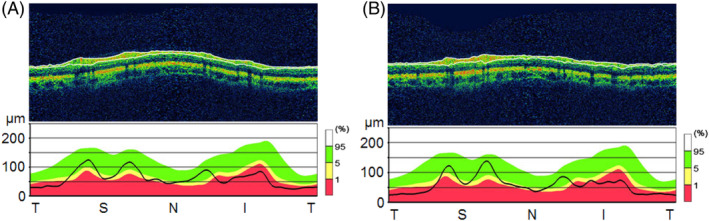
Optical examination in patient 1 after 36 months of CoQ 10 supplementation. Optical coherence tomography (OCT) associated with an analysis of the retinal nerve fiber layer (RNFL). (A) Right eye. (B) Left eye. Minimal optic atrophy

Supplement treatment with Idebenone (hydrophilic short‐chain coenzyme Q 10 analogue) was started 3 months after the onset of optical symptoms. We administered 15 mg/kg/day in three divided doses. Prior to initiating Ibedenone, the VA was still unchanged, recorded at 20/100 for the right eye and 20/50 for the left. An optical examination revealed a persistent slight papilledema without optic atrophy. Goldmann visual field examination revealed a central scotoma. After 2 months of treatment, we observed an improvement in the VA, increasing to 20/32 for the right eye and 20/32 for the left. Optical examination revealed the papilledema to have disappeared, with onset of optic atrophy (Figure 2). This optic atrophy was the result of the initial optical fiber impairment. The central scotoma was still present in the visual field. After 13 months of treatment, when the patient was 18 years old, the optical examination was stable. The patient did not recover normal vision, still exhibiting a bilateral VA of 20/32 and persistent optic atrophy. Goldmann visual field exam revealed the central scotoma to have disappeared and a new manifestation of small paracentral scotomas. Optical examination performed after 2 years of treatment demonstrated an improved VA at 20/20 for the right eye and 20/25 + for the left. And after 3 years of treatment the VA was stable at 20/25 + for both eyes with a minimal optic atrophy (Figure 3). The deafness status had not changed since treatment initiation. Renal function remained stable and the neurological examination was still normal. No treatment side effects were observed.

At the time of writing, the patient exhibited normal psychomotor development and was looking for employment.

### 2.2 Patient 2

Patient 2, the little brother of patient 1, was born after a term pregnancy and normal delivery. Psychomotor development and growth were normal. Bilateral sensorineural deafness was diagnosed at the age of 4 years, requiring equipment at 6 years old.

Given the patient's development of deafness and family history, he was monitored nephrologically, with non‐nephrotic glomerular proteinuria revealed at 5 years old. Angiotensin‐converting enzyme (ACE) inhibitor treatment was initiated, consisting of Enalapril. The proteinuria then became nephrotic and a course of prednisone was started. His NS was resistant to steroids, renal biopsy revealed FSGS, and skin biopsy was normal. The anti‐proteinuric treatment was enhanced by increasing the Enalapril associated with angiotensin receptor antagonists (Losartan) and diuretics (hydrochlorothiazide), resulting in complete remission of the NS. At the time of publishing, the anti‐proteinuric treatment consisted of Enalapril combined with Losartan. The patient exhibited normal psychomotor development and was following a normal school curriculum.

Genetic analysis of patient 1 revealed primary Coenzyme Q10 deficiency caused by a *COQ6* mutation. Resulting from this finding, a genetic analysis was performed for patient 2, which found the same homozygote mutation. This mutation was present in the heterozygous state in the patients' healthy parents and sister. Following this, an optical examination was performed, revealing no abnormalities.

The patient was started on Idebenone treatment at 10 mg/kg/day at 7 years old. After 13 months of treatment, hearing loss was unchanged. Renal involvement remained stable with only Enalapril, demonstrated by negative proteinuria. He presented no new symptoms, notably neither ophthalmological nor neurological.

## 3 DISCUSSION

In this study, we report on the cases of two brothers from a consanguineous Turkish couple who both presented a primary CoQ10 deficiency caused by a *COQ6* mutation. They presented with SRNS, bilateral sensorineural deafness, and optical involvement, consisting of optic neuropathy in patient 1. Idebenone treatment appeared to be effective in resolving optical impairment.

CoQ10, also known as ubiquinone, is an essential component of the mitochondrial electron transport chain and one of the most potent lipophilic antioxidants. CoQ10 operates as a redox carrier in the mitochondrial respiratory chain, shuttling electrons from respiratory chain complexes I (NADPH dehydrogenase) and II (succinate dehydrogenase) to complex III (ubiquinol cytochrome‐c reductase). CoQ10 has also been implicated in the inhibition of apoptosis by its prevention of inner mitochondrial membrane collapse.^3^, ^4^

The biochemical pathway of CoQ10 biosynthesis is complex and has yet to be fully elucidated, though we know that it requires at least 13 genes. Mutations in these genes cause primary CoQ10 deficiency, with ubiquinone biosynthetic gene mutations not only causing CoQ10 deficiency, but also having been implicated in monogenic mitochondriopathy.^3^, ^4^, ^5^

CoQ10 deficiency is a biochemical occurrence that was first described in 1989 and has since been associated with a wide variety of clinical phenotypes.^6^ Primary deficiencies are highly heterogeneous, both clinically and genetically, and are usually transmitted as autosomal recessive traits. Primary CoQ10 deficiency is generally characterized by clinical symptoms involving the central nervous system, skeletal muscle, and peripheral nerves^7^, ^8^, ^9^ with renal impairment being the primary complication in certain subtypes. *COQ2*, *COQ6*, *PDSS2*, and *ADCK4* (renamed to *COQ8B*) mutations are responsible for SRNS, associated with different extra‐renal disorders depending on the gene mutated (2, 3, 7, 8, 9, 10, 11, 12, 13, 14) (Table 1). *COQ9* mutations are responsible for renal tubulopathy with extra‐renal manifestations.^5^ Some cases of isolated SRNS have been described in patients with *COQ2* and *COQ8B* mutations.^3^, ^7^, ^9^, ^10^, ^11^ The link between CoQ10 and renal disease was first established in 2000 by Rotig et al when three siblings were diagnosed with a complex clinical syndrome characterized by progressive encephalomyopathy and SRNS. At this time, the mutation was unknown.^15^

**Table 1:** Renal, extra‐renal involvement and response after treatment in primary coenzyme Q10 deficiency in literature

Gene	Cases (family)	Clinical symptoms	Renal histology	Outcome after or without CoQ10 treatment	Ref
*PDSS2*	3 (1)	NS, encephalopathy, optic atrophy, sensorineural deafness, hypertrophic cardiomyopathy	ND	2 patients treated with neurological and ophthalmic improvement 1 patient died without treatment at 8 months	15
*PDSS2*	1 (1)	NS, seizures, hypotonia	ND	Treated, died at 8 months	13
*COQ2*	2 (1)	Isolated SRNS for the sister SRNS, mild psychomotor delay, optic atrophy for the brother	FSGS	2 patients treated with recovery of renal function, decreased of proteinuria for the sister; neurological improvement but renal transplant at 3 years for the brother	8‐9
*COQ2*	2 (2)	Isolated SRNS for the first patient NS, encephalopathy, respiratory failure for the second	Collapsing glomerulopathy	First patient was treated and remained stable Second patient died without treatment at 6 months	11
*COQ2*	2 (1)	NS, liver failure, seizures, pancytopenia, insulin‐dependent diabetes	ND	Died at 1 and 12 days without treatment	10
*COQ2*	2 (1)	Respiratory failure seizures, hypotonia	ND	Died at 5 and 6 months without treatment	16
*COQ2*	4 (2)	Multiple‐system atrophy, retinitis Pigmentosa	ND	2 patients alive without treatment, 2 patients died without treatment	17
*COQ2*	1 (1)	NS, Myoclonic epilepsy, hypertrophic cardiomyopathy	FSGS	Treated, died at 5 months	12
*COQ2*	1(1)	Hypertrophic cardiomyopathy, encephalopathy, respiratory failure	ND	Died at 2 months without treatment	18
*COQ9*	1 (1)	Renal tubulopathy, cardiomyopathy, neurological impairment	ND	Treated, died at 2 years	5
*ADCK4*	15 (8)	SRNS, developmental delay for one patient	FSGS	1 patient treated with decreased of proteinuria	14
*COQ6*	11 (5)	SRNS, sensorineural deafness, neurological impairment	FSGS, DMS	3 patients treated with decreased of proteinuria or deafness improvement	2
*COQ6*	2 (1)	SRNS, sensorineural deafness and optic atrophy for one patient	FSGS	Both treated, ophthalmic improvement	Our Case reports

*ADCK4,* aarF domain containing kinase 4; *COQ2,* coenzyme Q2 4‐hydroxybenzoate polyprenyltransferase; *COQ9*, Ubiquinone bisynthesis protein coenzyme Q9; *COQ6*, coenzyme Q 10 biosynthesis monooxygenase 6; DMS, diffuse mesangial sclerosis; FSGS, focal segmental glomerulosclerosis; ND, no data available; NS, néphrotic syndrome; *PDSS2,* prenyl (decaprenyl) diphosphate synthase subunit 2; Ref, references; SRNS, steroid resistant nephrotic syndrome.

*COQ2* mutation‐related nephropathy was the first to be described, reported in 2005. Individuals with *COQ2* mutations presented with phenotypes ranging from isolated NS to neonatal multisystem disorder with encephalomyopathy and renal involvement, as well as a recently described case of multiple‐system atrophy. Most affected individuals exhibit seizures, motor or mental retardation or hypotonia, optic atrophy, and SRNS. To date, *COQ2* mutations have been identified in 14 patients from nine different families. Five underwent kidney biopsy, which revealed FSGS for three and a collapsing glomerulopathy for the remaining two^5^, ^7^, ^9^, ^11^, ^13^, ^14^, ^16^, ^17^, ^18^ (Table 1).

In addition, *PDSS2* mutations have also been implicated in monogenic SRNS. The patients described in 2000 by Rotig et al were affected by a *PDSS2* mutation.^15^, ^19^ Moreover, Lopez et al reported a child with *PDSS2* mutation who developed Leigh syndrome with drug‐resistant seizures, SRNS, and cortical blindness.^12^

In 2009, Duncan et al reported, in a young boy, mutations of another gene, *CoQ9*, required for the biosynthesis of CoQ10. He presented with cardiomyopathy, renal tubulopathy, and neurological symptoms, later developing severe seizures and dying at 2 years old.^5^

Furthermore, autosomal recessive *COQ6* mutations have recently been identified in 11 individuals from five Lebanese and Turkish families.^2^ This gene encodes monooxygenase‐6. Coenzyme Q10 monooxygenase 6 (COQ6) is required for the biosynthesis of Coenzyme Q10 and is thought to catalyze one or more ring hydroxylation steps. All 11 affected individuals presented with SRNS. They each exhibited proteinuria at a median age of 1.2 years (range: 0.2‐6.4 years) and had all progressed to ESRF by a median age of 1.7 years (range: 0.4‐9.3 years), with five dying in early childhood (median age: 5.0 years). Renal biopsy revealed FSGS in seven cases and diffuse mesangial sclerosis in one. One patient presented with seizures, another exhibited white matter abnormalities and seizures and died of multi‐organ failure in sepsis, and two other individuals exhibited ataxia and facial dysmorphism. Nine of the 11 patients had sensorineural deafness. The *COQ6* mutations carried by our patients had already been described by Heeringa et al in Turkish families, and the renal presentation and deafness we observed were similar to those of that study. However, our study's two brothers had still not developed neurological involvement at the ages of 18 and 8 years, respectively. Also, unlike our patient, none of the patients of the Heeringa cohort presented with optic atrophy. The ophthalmic symptoms of our patient appeared late, however; 10 years after the deafness and ESRF, and the Heeringa study involved only short‐term follow up. Our study therefore illustrates the clinical heterogeneity of primary CoQ10 deficiency.

In a more recent study conducted in 2013, Ashraf et al identified recessive mutations in *COQ8B* that cause primary Coenzyme Q10 deficiency. This is a novel single‐gene cause of SRNS. All 15 affected individuals of the eight families studied exhibited SRNS. All manifested with proteinuria at a median age of 16 years (range: <1‐21 years) and progressed to ESRF by a median age of 15.3 years (range: 7‐23 years). Renal biopsy revealed FSGS in most cases and one patient manifested neurological involvement with psychomotor retardation.^10^

While most forms of monogenic childhood NS are characterized by a lack of response to therapy, some symptoms of primary CoQ10 deficiencies can respond to specific therapy. No evidence‐based study regarding dosage has yet been performed, however, and most patients receive empirical doses of CoQ10 ranging from 5 to 30 mg/kg per day.^3^, ^8^, ^20^, ^21^, ^22^ Furthermore, no information on the pharmacokinetics of CoQ10 in CoQ10‐deficient patients is currently available.

Response to replacement therapy is variable in primary CoQ10 deficiency. Many patients with encephalomyopathy have been shown to respond to CoQ10 supplement, demonstrating significant improvement in neurological symptoms.^8^, ^20^, ^23^, ^24^ Response to CoQ10 therapy in patients with SRNS is, however, highly variable (Table 1).

Some studies have reported no positive change in symptoms following oral CoQ10 supplementation. Scalais et al described a patient with *COQ2* mutation who presented with hypertrophic cardiomyopathy, epilepsy, and NS. Despite receiving Coenzyme Q10 supplement, administered at 30 mg/kg/day, the patient passed away at 5 months old.^14^ One patient with *PDSS2* mutation‐related deficiency with SRNS and drug‐resistant seizures received oral CoQ10 supplement and died at 8 months old.^12^ The individual with *COQ9* mutation was treated with CoQ10 supplement at 300 mg/day and died at 2 years of age.^5^

Rotig et al were the first to report a family in which CoQ10 deficiency caused by *PDSS2* mutation responded to CoQ10 supplement, their study involving three affected siblings.^15^, ^19^ Two siblings developed bilateral sensorineural deafness, optical and neurological symptoms, and NS resulting in terminal renal failure and required transplantation. The third sibling had a more severe disease course and died at 8 years old following rapid neurological deterioration. The two surviving children were treated with oral CoQ10 5 mg/kg per day, which resulted in substantial improvement in their neurological and ophthalmic conditions. The treatment did not, however, improve their renal function, perhaps owing to the patients already having advanced kidney disease.

In 2005, Salvati et al described another family with CoQ10 deficiency caused by *COQ2* mutation, presenting with infantile encephalomyopathy and nephropathy. The boy exhibited encephalomyopathy, optic atrophy, and SRNS. After receiving CoQ10 supplement at 22 months old, the child's neurological manifestations improved dramatically, though there was no change in renal function owing to an already‐advanced chronic renal failure. He underwent renal transplantation at 3 years old. His sister presented with SRNS without neurological symptoms. After 3 weeks of CoQ10 supplement at 30 mg/kg per day, her proteinuria levels were reduced and renal function recovered. No neurological impairment appeared. Early administration of CoQ10 was a crucial factor in the resolution of renal symptoms and preventing neurological damage in this patient.^7^, ^9^

Diomedi‐Camassei et al reported the case of an 18‐month‐old boy with severe SRNS resulting in terminal renal failure. His neurological examination remained completely normal when receiving CoQ10 supplement (30 mg/kg/day) for 8 months of follow up.^11^

Only one individual with *COQ8B* mutation, presenting with renal and neurological impairment, has been treated with oral CoQ10 (15 mg/kg/day) for over 4 years. This treatment resulted in significantly decreased proteinuria.^10^

Regarding the patients with *COQ6* mutation in Heeringa et al cohort, three received oral CoQ10 replacement therapy. The first patient was treated at 2 months old with CoQ10 at 30 mg/kg/day, associated with ACE inhibitors, while exhibiting non‐nephrotic proteinuria. After 13 months of treatment, we noted a decrease in proteinuria and return to normal renal function, yet an onset of deafness at 10 months old. For the second patient, his sister, who presented with bilateral deafness and ESRF, CoQ10 treatment consisting of 100 mg per day resulted in a substantial improvement of her deafness. The third patient exhibited bilateral deafness and SRNS, with cyclosporin A treatment inducing partial remission. After 2 months of treatment with CoQ10, his proteinuria levels decreased but hearing was not improved.^2^

Given the previous reports, we administered Idebenone in both of our patients. Idebenone is a hydrophilic short‐chain coenzyme Q 10 analogue. We choose to give Idebenone because it's the choice treatment in the Leber optic neuropathy. At this time no ophthalmic involvement has not been described yet in CoQ10 deficiency. So we preferred choose a treatment with efficacy proved on optic involvement.^25^, ^26^ Idebenone was given at 15 mg/kg/day in patient 1, resulting in an improvement in his ophthalmic impairment. After 2 months of Idebenone therapy, his VA improved, though optical examination revealed a bilateral optic atrophy probably caused by bilateral papilledema. After 13 months of supplement, the optical results remained stable, and after 36 months VA was found to have improved, with minimal optic atrophy. Idebenone treatment did not, however, improve the hearing loss. Renal function remained stable and the neurological findings were unchanged throughout. Patient 2 had received Idebenone since 24 months. No change in hearing loss was observed, the proteinuria was still negative, and the renal function remained stable. Replacement therapy could potentially prevent optical and renal disease in this patient, along with other symptoms in both patients, such as neurological disorders. This case report brings evidence of usefulness of the Idebenone. Maybe Idebenone could even be better for patients with defects with CoQ10 compared to CoQ10.

## 4 CONCLUSION

In conclusion, CoQ10 deficiency caused by *COQ6* mutations is a rare cause of SRNS with variable neurological, renal, hearing, and optical impairment. CoQ10 deficiencies are clinically and genetically heterogeneous diseases. Mitochondrial dysfunction secondary to CoQ10 deficiency should always be suspected and investigated in patients with SRNS and extra‐renal symptoms. Early recognition of this genetic entity of SRNS is a crucial diagnostic tool, as this rare disorder can be successfully treated by CoQ10 supplement. All cases of primary CoQ10 deficiency should be treated at an early stage to limit the progression of lesions and prevent the emergence of new symptoms. In this case report we used Idebenone, a CoQ10 analogue, and the patients seemed to response. Efficacy and perhaps superiority of Idebenone over coenzyme Q 10 needs to be confirmed by a clinical study.

## CONFLICT OF INTEREST

The authors have no conflict of interest to disclose.

## INFORMED CONSENT

All procedures followed were in accordance with the ethical standards of the responsible committee on human experimentation (institutional and national) and with the Helsinki Declaration of 1975, as revised in 2000 (5). Informed consent was obtained from all patients for being included in the study.

## ANIMAL RIGHTS

This article does not contain any studies with human or animal subjects performed by the any of the authors.

## FINANCIAL DISCLOSURE

The authors have no financial relationships relevant to this article disclose.
